# Childhood traumatization among elite athletes – preliminary study

**DOI:** 10.1192/j.eurpsy.2025.1953

**Published:** 2025-08-26

**Authors:** N. M. Szeifert, B. Sebok, A. Lenart, X. Gonda

**Affiliations:** 1 Department of Sports Medicine, Semmelweis University; 2 National Institute of Sports Medicine; 3 Eotvos Lorand University, Doctoral School of Psychology; 4 School of PhD Studies Workgroup for Science Management, Semmelweis University; 5 Dr. Manninger Jenő Trauma Center; 6 Department of Psychology and Sport Psychology, Hungarian University of Sports Science; 7 Department of Psychiatry and Psychotherapy, Semmelweis University, Budapest, Hungary

## Abstract

**Introduction:**

Achieving excellent sport performance is based both on physical strength and mental endurance. Research demonstrated that children experiencing early maltreatment and traumatization are at an increased risk of high-risk behaviours, bullying, and mental and somatic disorders lasting into adulthood effecting academic and sport performance, relations and life management. Research found that individuals with a history of trauma had a significantly lower chance of being diagnosed with depression or anxiety if they had participated in team sports as adolescents. Also later in life, sport is very beneficial to cope with stressful life events.

**Objectives:**

We aimed to explore occurrence of early traumas in elite athletes and a control group using the Childhood Trauma Questionnaire (CTQ) and the Early Trauma Inventory Self Report-Short Form (ETISR-SF).

**Methods:**

The sample included 57 subjects, 50.9% elite athletes and 49.1% leisure time athletes (controls). Participants (64.9% males, 35.1% females) provided demographic data (age 18-58 years, mean age = 31.96 years, SD = 8.920) and completed the ETISR-SF and the Childhood Trauma Questionnaire-Short Form (CTQ-SF). Analysis of Fisher’s Exact Test was performed for evaluating the occurrence of early traumatization.

**Results:**

The ETISR-SF and CTQ subscales scales did not show significant difference in any type of traumas in elite athletes compared to the control group (general traumas p=0.092, emotional abuse p=1.000, physical abuse p=0.592, sexual abuse p=0.297). Occurrence of each trauma type in the two groups were as follows. Elite athletes: sexual trauma (10.34%), physical trauma (55.17%), emotional trauma (44.83%), general trauma (55.17%); Controls: sexual trauma (21.43%), physical trauma (64.29%), emotional trauma (46.43%), general traumas (78.57%).

**Conclusions:**

The occurrence of different type of trauma in our sample is in line with the literature. Physical and emotional abuse among athletes is high. Presumably, those athletes perform better who experience less trauma. The best way to produce resilient athletes is to provide a challenging and supportive sport environment, where athletes feel physically and psychologically safe in their experiences of success and failure.

**Disclosure of Interest:**

None Declared

